# Cell cycle inhibition reduces inflammatory responses, neuronal loss, and cognitive deficits induced by hypobaria exposure following traumatic brain injury

**DOI:** 10.1186/s12974-016-0769-2

**Published:** 2016-12-01

**Authors:** Jacob W. Skovira, Junfang Wu, Jessica J. Matyas, Alok Kumar, Marie Hanscom, Shruti V. Kabadi, Raymond Fang, Alan I. Faden

**Affiliations:** 1 Department of Anesthesiology and Center for Shock, Trauma and Anesthesiology Research (STAR), University of Maryland School of Medicine, Baltimore, MD 21201 USA; 2 Research Division Pharmacology Branch, United States Army Medical Research Institute of Chemical Defense, Aberdeen Proving Ground, Aberdeen, MD 21010 USA; 3 Program in Trauma, Center for the Sustainment of Trauma and Readiness Skills (C-STARS), University of Maryland School of Medicine, Baltimore, MD 21201 USA

**Keywords:** Traumatic brain injury, Inflammation, Neuronal cell death, Aeromedical evacuation, Hypobaria

## Abstract

**Background:**

Traumatic brain injury (TBI) patients in military settings can be exposed to prolonged periods of hypobaria (HB) during aeromedical evacuation. Hypobaric exposure, even with supplemental oxygen to prevent hypoxia, worsens outcome after experimental TBI, in part by increasing neuroinflammation. Cell cycle activation (CCA) after TBI has been implicated as a mechanism contributing to both post-traumatic cell death and neuroinflammation. Here, we examined whether hypobaric exposure in rats subjected to TBI increases CCA and microglial activation in the brain, as compared to TBI alone, and to evaluate the ability of a cyclin-dependent kinase (CDK) inhibitor (CR8) to reduce such changes and improve behavioral outcomes.

**Methods:**

Adult male Sprague Dawley rats were subjected to fluid percussion-induced injury, and HB exposure was performed at 6 h after TBI. Western blot and immunohistochemistry (IHC) were used to assess cell cycle-related protein expression and inflammation at 1 and 30 days after injury. CR8 was administered intraperitoneally at 3 h post-injury; chronic functional recovery and histological changes were assessed.

**Results:**

Post-traumatic hypobaric exposure increased upregulation of cell cycle-related proteins (cyclin D1, proliferating cell nuclear antigen, and CDK4) and microglial/macrophage activation in the ipsilateral cortex at day 1 post-injury as compared to TBI alone. Increased immunoreactivity of cell cycle proteins, as well as numbers of Iba-1^+^ and GFAP^+^ cells in both the ipsilateral cortex and hippocampus were found at day 30 post-injury. TBI/HB significantly increased the numbers of NADPH oxidase 2 (gp91^phox^) enzyme-expressing cells that were co-localized with Iba-1^+^. Each of these changes was significantly reduced by the administration of CR8. Unbiased stereological assessment showed significantly decreased numbers of microglia displaying the highly activated phenotype in the ipsilateral cortex of TBI/HB/CR8 rats compared with TBI/HB/Veh rats. Moreover, treatment with this CDK inhibitor also significantly improved spatial and retention memory and reduced lesion volume and hippocampal neuronal cell loss.

**Conclusions:**

HB exposure following TBI increases CCA, neuroinflammation, and associated neuronal cell loss. These changes and post-traumatic cognitive deficits are reduced by CDK inhibition; such drugs may therefore serve to protect TBI patients requiring aeromedical evacuation.

## Background

Traumatic brain injury (TBI) is a major cause of morbidity and mortality in civilian populations [1] and has been a serious concern for US military forces, where the number of cases has nearly tripled over the last decade [2]. TBI casualties are moved from the battlefield to the appropriate level of care through the military aeromedical evacuation (AE) system [3]. During transport, patients can be exposed to long periods of hypobaria (HB), as military flights are often pressurized only to 8000 ft, substantially different from commercial air travel [3, 4]. It has been recently shown in a rat TBI model that hypobaria during simulated AE worsens cognitive and pathological outcomes [5]; this report and an earlier one using a mouse TBI model also suggest that hypobaria can increase post-traumatic inflammatory responses [6].

TBI-related neuropathology reflects both direct mechanical damage (primary injury) and delayed induced molecular and cellular cascades (secondary injury)—leading to neuronal cell death, axonal disruption, demyelination, astrogliosis, and inflammation [7]. Cell cycle activation (CCA) occurs after TBI in both neurons and glial cells and contributes to secondary injury [8–10]. In post-mitotic cells such as neurons, CCA contributes to programmed cell death. In glia, CCA induces astrocyte and microglial proliferation/reactivation, leading to astroglial scar formation, release of pro-inflammatory cytokines and reactive oxygen species (ROS), and ultimately neuronal degeneration [8–10]. Administration of cell cycle inhibitors after TBI increases neuronal survival and reduces both microglial and astroglial activation; the latter includes multiple studies utilizing the rat LFP injury model [11–15].

TBI-induced neuroinflammation appears to play a pivotal role in secondary injury severity and progression. Although the neuroinflammatory response to injury may have either beneficial or detrimental actions [16], both pre-clinical and clinical studies show that chronic microglial activation after TBI contributes to both progressive neurodegeneration and related neurological deficits [17–19]. As sustained post-traumatic CCA appears to contribute to chronic neuroinflammation, this study was designed to evaluate whether HB following TBI increases both CCA and related neuroinflammation and whether CCA inhibition can limit these harmful consequences of hypobaric exposure and reduce cognitive dysfunction.

## Methods

### Animals

Male Sprague Dawley rats (Harlan Labs, Frederick, MD) weighing 325 g (±25 g) were utilized for this study. Animals were fed a standard laboratory diet with food and water ad libitum. All procedures and experiments were carried out in accordance with protocols approved by the Animal Care and Use Committee at the University of Maryland and the United States Air Force.

### Micro-fluid percussion and hypobaric animal experiments

Rats were anesthetized with isoflurane (4% induction, 2% maintenance), and a 5-mm craniotomy was made over the left parietal cortex midway between the lambda and bregma as previously described [5, 20]. Using our custom micro-fluid percussion (FP) device, a 1.5–1.9-atmosphere (atm) pressure was used to produce a mild injury with regard to neurologic and histologic deficits [20]. Sham animals underwent the same procedures without injury. Hypobaria was induced using a steel cylindrical chamber with interior dimensions of 46 cm wide and 112 cm long equipped with internal temperature, oxygen, carbon dioxide, and pressure gauges and connected to a vacuum pump. Animals were placed into the chamber in their home cages with access to water and food to reduce stress from acclimation to the HB chamber. Multiple animals in various groups were randomly exposed simultaneously. The chamber was de-pressurized over 30 min to reach 568 mmHg (=8000 ft. altitude)—approximating the cabin pressure during military AE with cruising altitudes of 30,000–40,000 ft. To account for the mean oxygen saturation decrease of 5.5% experienced at this pressure, 28% O_2_ was continuously delivered to the chamber to maintain pO_2_ at sea level despite the drop in atmospheric pressure. Chamber gases were continuously monitored to validate concentration of O_2_ delivered, as well as to verify that CO_2_ was not accumulating in the chamber. At 5.5 h of “flight,” the chamber was re-pressurized over 30 min to 1 atm (765 mmHg), and the animals were then removed. Interior chamber temperature was monitored continuously and maintained at 22 ± 2 °C.

### Experimental procedure

**Fig. 1:**
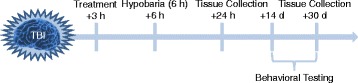
Experimental timeline for evaluating CR8 treatment on simulated AE-induced cell cycle activation and inflammatory responses following TBI

An inhibitor of cell cycle activation—(2-(R)-(1-ethyl-2-hydroxyethylamino)-6-(4-(2-pyridyl)benzyl)-9-isopropylpurine trihydrochloride (CR8, Tocris Bioscience, Minneapolis, MN), was evaluated for its effects on cellular inflammatory reactions (microglial and astrocyte activation) as well as on histologic and neurologic outcome after TBI. For tissue collection experiments, male rats were randomized to one of four groups: sham injury, TBI alone, TBI + HB + vehicle (Veh), and TBI + HB + CR8 (Fig. 1). For behavioral analysis and stereology experiments, male rats were randomized to one of three groups: sham injury, TBI + HB + Veh, and TBI + HB + CR8 (Fig. 1). We have previously reported a TBI-alone group for behavioral analysis [5]. Animals in the treatment groups received a dose of CR8 (5 mg/kg in saline, IP) or an equal volume of vehicle (saline) 3 h following the induction of a TBI. Six hours after the induction of TBI (1.5–1.9 atm), the animals were exposed to HB for 6 h at 0.75 atm. This dose and timing of administration was based on previous studies using this compound in experimental animal models of TBI—which have shown neuroprotection by limiting microglial activation, astrocytosis, and neuronal loss [5, 15]. Behavioral tests were conducted over 30 days post-HB. This experimental timeline was chosen to be consistent and comparable to established procedures for accurate assessment of behavioral and histological outcomes following TBI [5, 15]. All behavioral tests were conducted by an experimenter blinded to the experimental groups. Behavioral testing included Morris water maze tests for learning and memory (post-HB days 14–18), novel object recognition test for retention memory (post-HB day 21), and the forced swim test (post-HB day 26) for depressive-like behaviors. Brains were collected at 24 h post-injury or on post-HB day 30 for pathologic or immunohistochemical analysis. The number of rats in each group or subgroup is indicated in Table 1.

**Table 1:** Definition of the groups

Groups/rats #	Functional assessment	Outcome measures in subgroups (subgroups, randomly selected)
24 h TBI		
Sham	7	WB
TBI	9	WB
TBI/Veh/HB	10	WB
TBI/CR8/HB	7	WB
30 days TBI		
Sham	16	Behavioral tests, IHC
TBI	6	IHC
TBI/Veh/HB	14	Behavioral tests, IHC, lesion volume, stereology
TBI/CR8/HB	15	Behavioral tests, IHC, lesion volume, stereology

*TBI* traumatic brain injury, *WB* western blot, *HB* hypobaria, *Veh* vehicle, *IHC* immunohistochemistry

### Tissue collection and western blot

At 24 h post-injury, rats were anesthetized with sodium pentobarbital (100 mg/kg, IP). A blunt 21-gauge needle connected to a peristaltic pump (Harvard Apparatus, Holliston, MA) primed with 0.9% sodium chloride (saline) was pierced through the left lateral ventricle and inserted diagonally into the ascending aorta. An incision was then made in the right atrium to allow the fluid to flow through. The brain was perfused with saline at a rate of 50 ml/min for 10 min before being removed. A 5-mm area surrounding the lesion epicenter on the ipsilateral cortex was rapidly dissected placed in a 1-ml microcentrifuge tube and flash frozen with liquid nitrogen. Frozen tissue samples were stored at −80 °C prior to analysis.

For all immunoblot samples, the cortical tissue was homogenized in radioimmunoprecipitation assay (RIPA) buffer and centrifuged at 15,000 rpm for 15 min at 4 °C to isolate proteins, and the protein concentration was determined using the Pierce BCA Protein Assay kit (Thermo Scientific, Rockford, IL). Twenty-five microgram of protein was run on sodium dodecyl sulfate (SDS) polyacrylamide gel electrophoresis and transferred onto nitrocellulose membrane (*n* = 6–10/group). The blots were probed with antibodies against cyclin-dependent kinase (CDK)4 (1:1000, Santa Cruz Biotechnology, Inc., Santa Cruz, CA); cyclin D1 (1:500, Santa Cruz Biotechnology, Inc., Santa Cruz, CA); proliferating cell nuclear antigen (PCNA; 1:500, Santa Cruz Biotechnology); and ionized calcium-binding adapter molecule 1 (Iba-1; 1:1000, Wako Chemicals, Richmond, VA), and GAPDH (1:2000; Sigma-Aldrich, St. Louis, MO) was used as an endogenous control. Immune complexes were detected with the appropriate horseradish peroxidase (HRP)-conjugated secondary antibodies (KPL, Inc., Gaithersburg, MD) and visualized using SuperSignal West Dura Extended Duration Substrate (Thermo Scientific, Rockford, IL). Chemiluminescence was captured on a Kodak Image Station 4000R station (Carestream Health, Rochester, NY), and protein bands were quantified by densitometric analysis using Carestream Molecular Imaging Software. The data presented reflect the intensity of the target protein band compared to the control and were normalized based on the intensity of the endogenous control for each sample.

### Functional assessment

#### Morris water maze

Spatial learning and memory were assessed using the acquisition paradigm of the Morris water maze (MWM) test as previously described [15]. A circular pool (1.5 m in diameter) was divided into four quadrants using computer-based AnyMaze video tracking system (Stoelting Co., Wood Dale, IL). Each rat was subjected to four trials to locate the hidden platform every day from post-HB days 14 to 17 (acquisition phase). Latency (seconds) to locate the hidden platform was measured, with a 90-s limit per trial, and swimming velocities assessed. Water maze search strategy analysis was also performed as previously described [15]. Reference memory was assessed by a probe trial carried out on post-HB day 18. A visual cue test was also performed on post-HB day 18.

#### Novel object recognition

Nonspatial retention and recognition memory was assessed by the novel object recognition test as previously described [5, 15]. On post-HB day 20, animals were placed into the open field and allowed to explore for 10 min each without any of the objects present for habituation and familiarization. On the testing day (post-HB day 21), two trials of 5 min each were performed. The first trial (training phase) involved placing identical square-shaped “old objects” in both zones of the open field. The second trial (testing phase) involved placing one square-shaped “old object” and one triangular-shaped “novel object” in the respective zones of the open field. The time that was spent exploring each object during both trials was recorded. In addition, time spent in the novel object and old object zones was analyzed and compared between groups separately. The cognitive outcomes were calculated as the “discrimination index” (D.I.) for the second trial using the following formula: % D.I. = (time spent exploring novel object / (total time spent exploring both objects)) × 100.

#### Forced swim test

The forced swim test was used to examine depressive-like behaviors [5, 15]. On post-HB day 26, rats were individually forced to swim inside a vertical plastic container (height 60 cm; diameter 25 cm) containing 30 cm of water for a time period of 6 min. The total duration of immobility (passive floating, slightly hunched, upright position, the head just above the surface) vs. struggle (diving, jumping, strongly moving all four limbs, scratching the walls) was recorded.

### Tissue processing, immunohistochemistry, image acquisition, and quantification

At 30 days after injury, rats were anesthetized and intracardially perfused with 200 ml of saline followed by 300 ml of 4% paraformaldehyde. The dissected brains were post-fixed for overnight and cryoprotected through a sucrose gradient. The coronal sections were cut, serially collected (3 × 60 μm followed by 3 × 20 μm sections) throughout the brain and mounted onto glass slides for histology and immunohistochemistry.

Standard fluorescent immunohistochemistry on serial, 20-μm-thick sections was performed as described previously [21]. The following primary antibodies were used: rabbit anti-CDK4 (1:500, Santa Cruz Biotechnology); mouse anti-cyclin D1 (1:500, Neomarker); rabbit anti-PCNA (1:500, Santa Cruz Biotechnology); rabbit or mouse anti-GFAP (1:1000, Chemicon); rabbit anti-Iba-1 (1:1000, Wako Chemicals); mouse anti-gp91^phox^ (1:500; BD Transduction Laboratories, Franklin Lakes, NJ); and galectin 3 (1:500, Santa Cruz Biotechnology). Fluorescent-conjugated secondary antibodies (1:1000, Alexa 488-conjugated goat anti-mouse or rabbit, Alexa Fluor 546 goat anti-mouse, Alexa Fluor 633 goat anti-mouse, Molecular Probes) were incubated with tissue sections for 1 h at room temperature. Counterstaining was performed with 4′,6-diamidino-2-phenylindole (DAPI) (1 μg/ml; Sigma-Aldrich). All immunohistological staining experiments were carried out with appropriate positive control tissue as well as primary/secondary-only negative controls.

For quantitative image analysis, images were acquired using a fluorescent Nikon Ti-E inverted microscope, at ×20 (CFI Plan APO VC 20× NA 0.75 WD 1 mm) magnification. Exposure times were kept constant for all sections in each experiment. Background for all images was subtracted using Elements. All images were quantified using Elements: nuclei were identified using Spot Detection algorithm based on DAPI staining; cells positive for any of the immunofluorescence markers were identified using Detect Regional Maxima or Detect Peaks algorithms, followed by global thresholding. The intensity of cyclin D1, PCNA, and CDK4 was normalized to the total area imaged. The number of positive cells was normalized to the total number of cells based on DAPI staining. All quantifications were performed in the ipsilateral cortex and hippocampus. For each experiment, data from all images from one region in each animal were summated and used for statistical analysis [22, 23]. At least 1000–2000 cells were quantified for each rat per area per experiment.

### Stereological quantification of microglial phenotypes in the ipsilateral cortex

Every fourth 60-μm brain section was immunostained for Iba-1 and DAB and analyzed using a Leica DM4000B microscope (Leica Micro-systems Inc., Buffalo Grove, IL, USA). The number of cortical microglia in either activated (hypertrophic and bushy) or resting (ramified) morphologic phenotypes were counted using the optical fractionator method with the Stereo Investigator software (MBF Biosciences) as described previously [5, 15]. Microglial phenotypic classification was based on the length and thickness of the projections, the number of branches, and the size of the cell body, as previously described [5]. The sampling region was between −2.04 and −4.56 mm from the bregma in the ipsilateral cortex with a dorsal depth of 2.0 mm from the surface. The volume of the region of interest was measured using the Cavalieri estimator method. The estimated number of microglia in each phenotypic class was divided by the volume of the region of interest to obtain cellular density expressed in counts per cubic millimeters (mm^3^).

### Lesion volume and neuronal survival in the hippocampal subregions

Sections were stained with cresyl violet (FD NeuroTechnologies, Baltimore, MD), dehydrated, and mounted for analysis. Lesion volume was quantified based on the Cavalieri method of unbiased stereology using Stereologer 2000 program software (Systems Planning and Analysis, Alexandria, VA). The lesion volume was quantified by outlining the missing tissue on the injured hemisphere using the Cavalieri estimator with a grid spacing of 0.1 mm. Every fourth 60-μm section between −2.04 and −4.56 mm from the bregma was analyzed beginning from a random start point.

The total number of surviving neurons in the cornus ammonis (CA)1, CA2, CA3, and dentate gyrus (DG) subregions of the hippocampus was assessed using the optical fractionator method [5, 15]. Every fourth 60-μm section between −2.04 and −4.56 mm from the bregma was analyzed, beginning from a random start point. The volume of each hippocampal subfield was measured using the Cavalieri estimator method. The estimated number of surviving neurons in each field was divided by the volume of the region of interest to obtain the neuronal cellular density, expressed as counts/mm^3^.

### Statistical analysis

Quantitative data were expressed as mean ± standard error of the mean (SEM). Analysis of histological data was conducted using a one-way ANOVA followed by the Student-Newman-Keuls post hoc test. Functional data (latency to find the platform in seconds) for the acquisition phase of the MWM were analyzed by repeated measure (trial over time) two-way ANOVA (TBI + Veh + HB vs. TBI + CR8 + HB) to determine the interactions of post-injury days and groups, followed by post hoc adjustments using the Student-Newman-Keuls test. As we are only interested in whether CR8 treatment improves outcomes over TBI + HB with vehicle treatment, further analysis of behavioral outcomes was conducted using a one-tailed unpaired Student’s *t* test to determine the differences between groups within each trial day. The comparison of search strategies during the final day of the trials of the MWM acquisition phase was analyzed using a chi-square test. As we are only interested in whether CR8 treatment improves outcomes over TBI + HB with vehicle treatment, all other analyses (MWM probe, novel object, forced swim, stereological assessments, lesion volume) were conducted using a one-tailed unpaired Student’s *t* test (TBI + Veh + HB vs. TBI + CR8 + HB). All tests were performed using either SigmaPlot 12 (Systat Software, San Jose, CA) or GraphPad Prism program; version 4.0 (GraphPad Software; San Diego, CA). A *p* value of less than 0.05 was considered statistically significant.

## Results

### Post-traumatic hypobaria exposure increases CCA as compared to TBI alone

**Fig. 2:**
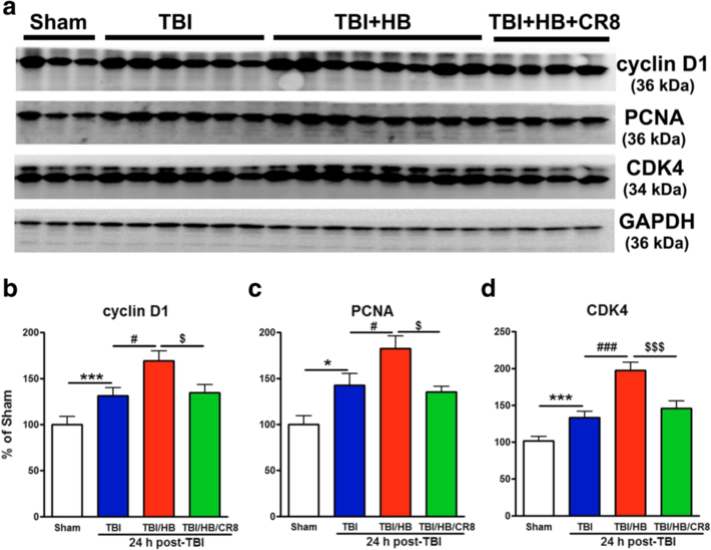
Hypobaria exposure increases markers of cell cycle activation at 24 h after TBI. **a** Representative immunoblots for cell cycle-related proteins (cyclin D1, PCNA, and CDK4) and the loading control (GAPDH). **b**–**d** Expression levels of cell cycle proteins were normalized by GAPDH, as estimated by optical density measurements, and expressed as a percentage of sham tissue. At 24 h post-injury, a significant increase in the protein expression of cyclin D1, PCNA, and CDK4 was observed in the TBI/no-HB exposure group in comparison to the sham injury group. HB exposure following TBI significantly increased the protein expression of all three markers in comparison to the TBI-alone group. A significant decrease in the protein expression of all three markers was observed in the CR8 treatment group in comparison to the TBI/Veh/HB group. *N* = 7 (sham), 9 (TBI), 10 (TBI/Veh/HB), 7 (TBI/CR8/HB). *p < 0.05, ***p < 0.001, TBI vs. sham injury; ^#^ *p* < 0.05, ^###^ *p* < 0.001, TBI/Veh/HB vs. TBI/no-HB; ^$^ *p* < 0.05, ^$$$^ *p* < 0.001, TBI/CR8/HB vs. TBI/Veh/HB

To evaluate the effect of HB on cell cycle activation, we first examined cell cycle pathway changes in the ipsilateral cortex at day 1 after TBI. Western blotting was performed for the markers of cell cycle-related proteins cyclin D1, PCNA, and CDK4 (Fig. 2). At 24 h post-injury, a significant increase in the protein expression of these markers was observed in the injured/no-HB exposure group (*n* = 9) in comparison to the sham injury group (*n* = 7, *p* < 0.001, TBI/no-HB vs. sham injury). HB exposure following TBI (*n* = 10) significantly further increased the protein expression of cyclin D1, PCNA, and CDK4 in comparison to the TBI/no-HB exposure group (*p* < 0.001 for CDK4, *p* < 0.05 for cyclin D1, TBI + HB vs. TBI/no-HB). In contrast, cell cycle inhibition by administration of CR8 (*n* = 7) limited the TBI + HB-induced increase in the expression of these cell cycle proteins at 1 day post-injury (*p* < 0.05 for cyclin D1 and PCNA, *p* < 0.001 for CDK4, TBI + Veh + HB vs. TBI + CR8 + HB).

**Fig. 3:**
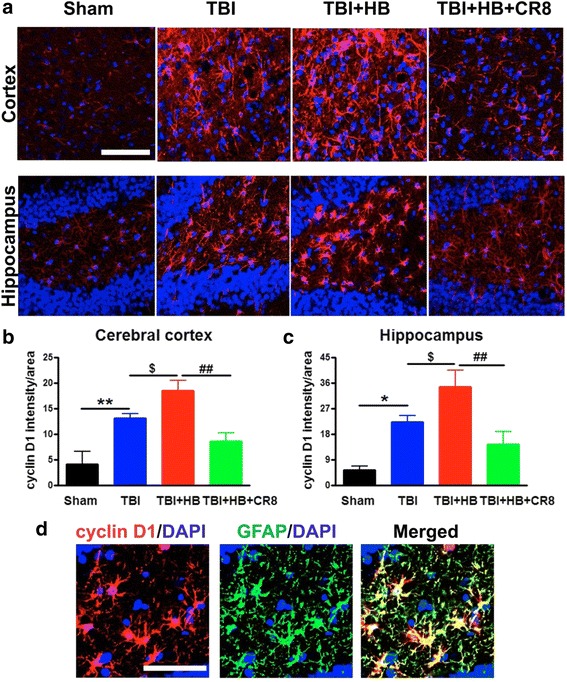
Hypobaria exposure upregulates the expression of cyclin D1 in the ipsilateral cortex and hippocampus at 30 days after TBI. **a** Representative immunofluorescent staining for cyclin D1 (*red*) and DAPI (*blue*). **b**, **c** Quantification of pixel intensity for cyclin D1 revealed significant increases in the TBI + HB group in contrast to the TBI-alone tissue. A significant decrease of cyclin D1 expression was observed in the CR8 treatment group in comparison to the TBI/Veh/HB group. *N* = 4 (sham), 6 (TBI), 6 (TBI/Veh/HB), 6 (TBI/CR8/HB). *p < 0.05, **p < 0.01, TBI vs. sham injury; ^$^ *p* < 0.05, TBI/Veh/HB vs. TBI; ^##^ *p* < 0.01, TBI/CR8/HB vs. TBI/Veh/HB. **d** Cyclin D1 (*red*) predominantly expressed by GFAP^+^ astrocytes (*green*; DAPI, *blue*). *Scale bar* = 50 μm in **a** and **d**

**Fig. 4:**
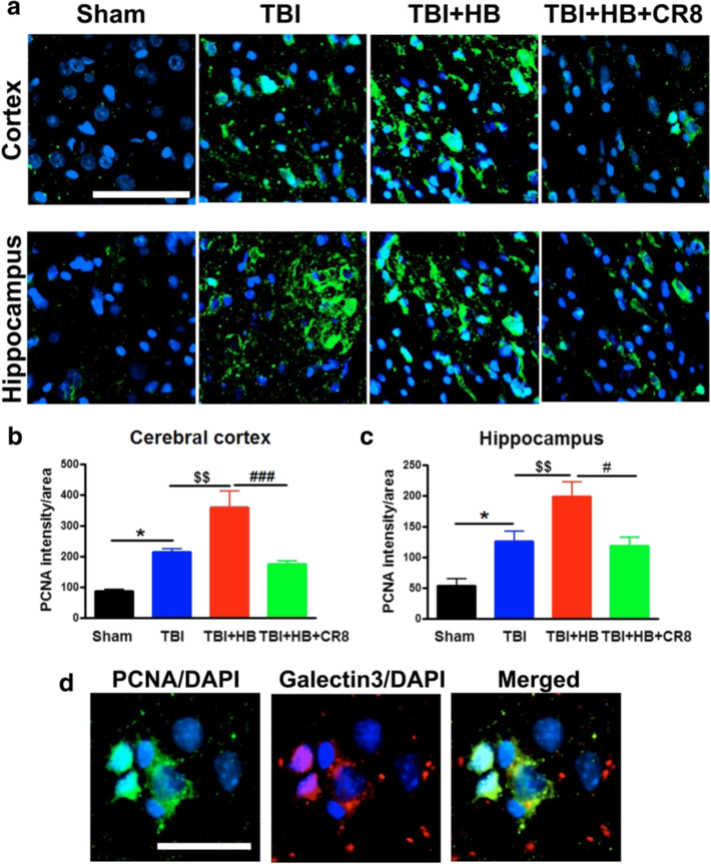
Hypobaria exposure upregulates the expression of PCNA in the ipsilateral cortex and hippocampus at 30 days after TBI. **a** Representative immunofluorescent staining for PCNA (*green*) and DAPI (*blue*). **b**, **c** Quantification of pixel intensity for PCNA revealed significant increases in the TBI + Veh + HB group in contrast to the TBI-alone tissue. A significant decrease of cyclin D1 expression was observed in the CR8 treatment group in comparison to the TBI/Veh/HB group. *N* = 4 (sham), 6 (TBI), 6 (TBI/Veh/HB), 6 (TBI/CR8/HB). *p < 0.05, TBI vs. sham injury; ^$$^ *p* < 0.01, TBI/Veh/HB vs. TBI; ^#^ *p* < 0.05, ^###^ *p* < 0.001, TBI/CR8/HB vs. TBI/Veh/HB. **d** Most of the PCNA^+^ cells (*green*) in the injured cortex were co-labeled with galectin 3-expressing microglia/macrophages (*red*; DAPI, *blue*). *Scale bar* = 50 μm in **a** and 25 μm in **d**

**Fig. 5:**
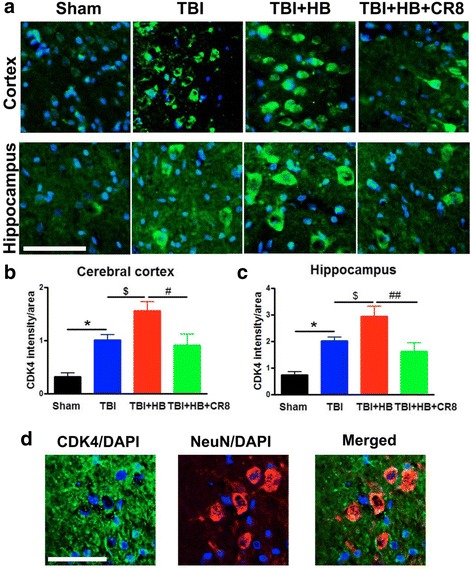
Hypobaria exposure upregulates the expression of CDK4 in the ipsilateral cortex and hippocampus at 30 days after TBI. **a** Representative immunofluorescent staining for CDK4 (*green*) and DAPI (*blue*). **b**, **c** Quantification of pixel intensity for CDK4 revealed significant increases in the TBI + Veh + HB group in contrast to the TBI-alone tissue. A significant decrease of CDK4 expression was observed in the CR8 treatment group in comparison to the TBI/Veh/HB group. *N* = 4 (sham), 6 (TBI), 6 (TBI/Veh/HB), 6 (TBI/CR8/HB). *p < 0.05, TBI vs. sham injury; ^$^ *p* < 0.05, TBI/CR8/HB vs. TBI; ^#^ *p* < 0.05, ^###^ *p* < 0.001, TBI/HB/CR8 vs. TBI/Veh/HB. **d** Some of the CDK4^+^ cells (*green*) in the injured cortex were co-labeled with NeuN-expressing neurons (*red*; DAPI, *blue*). *Scale bar* = 50 μm in **a** and 25 μm in **d**

We also examined immunoreactivity of cyclin D1, PCNA, and CDK4 in the ipsilateral cortex and hippocampus from sham (*n* = 4), TBI alone (*n* = 6), TBI + Veh + HB (*n* = 6), and TBI + CR8 + HB (*n* = 6) rats at 30 days post-injury. Quantification of pixel intensity for these markers showed significant increases in the TBI + Veh + HB group in contrast to the TBI-alone tissue (Figs. 3, 4, and 5). Cyclin D1 was predominantly expressed by GFAP^+^ astrocytes (Fig. 3d). Most of the PCNA^+^ cells in the injured cortex were co-labeled with galectin 3-expressing microglia/macrophages (Fig. 4d). Some of the CDK4^+^ cells displayed neuronal morphology and co-labeled with NeuN (Fig. 5a, d). In addition, CDK4 was also expressed by astrocytes (data not shown). The post-traumatic upregulation of these proteins was attenuated by CR8 treatment.

### Cell cycle inhibition reduces microglial activation and astrogliosis induced by hypobaria exposure following TBI

**Fig. 6:**
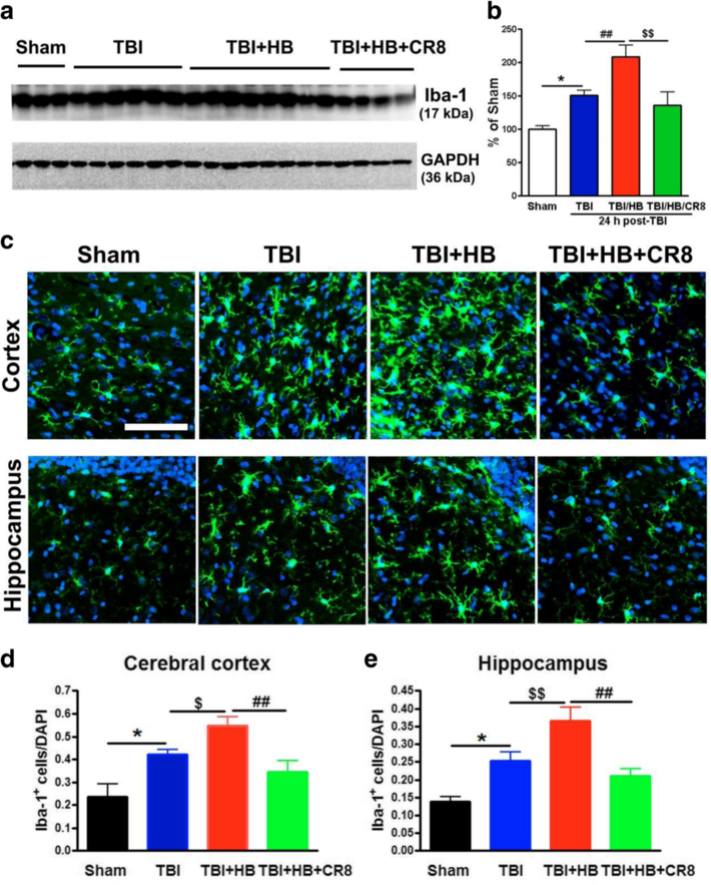
Cell cycle inhibition reduces protein expression of microglial/macrophages marker Iba-1 induced by hypobaria exposure after TBI. **a** Representative immunoblots for Iba-1 and the loading control (GAPDH). **b** Quantification of the expression levels of Iba-1 protein revealed significant increases in the TBI + Veh + HB group in contrast to the TBI-alone group in the ipsilateral cortex at day 1 after TBI. A significant decrease of Iba-1 expression was observed in the CR8 treatment group in comparison to the TBI/Veh/HB group. *N* = 7 (sham), 9 (TBI), 10 (TBI//Veh/HB), 7 (TBI/CR8/HB). *p < 0.05, TBI vs. sham injury; ^##^ *p* < 0.01, TBI/Veh/HB vs. TBI no HB; ^$$^ *p* < 0.01, TBI/CR8/HB vs. TBI/Veh/HB. **c** Representative immunofluorescent staining for Iba-1 (*green*) at 30 days post-injury and DAPI (*blue*). **d**, **e** Quantification of Iba-1^+^ cells revealed significant increases in the TBI + Veh + HB group in contrast to the TBI-alone group. A significant decrease of Iba-1^+^ cells was observed in the CR8 treatment group in comparison to the TBI/Veh/HB group. *N* = 4 (sham), 6 (TBI), 6 (TBI/Veh/HB), 6 (TBI/CR8/HB). *p < 0.05, TBI vs. sham injury; ^$^ *p* < 0.05, ^$$^ *p* < 0.01, TBI/Veh/HB vs. TBI; ^###^ *p* < 0.001, TBI/CR8/HB vs. TBI/Veh/HB. *Scale bar* = 50 μm

To examine whether HB-induced activation of microglia and astrocytes were attenuated by inhibiting CCA, rats were treated with CR8 or saline by ip injection at 3 h post injury and the ipsilateral cerebral cortical tissue was collected at 24 h after TBI. Quantitative analysis of western blots showed that Iba-1 expression in TBI (*n* = 9) or TBI + Veh + HB (*n* = 10) groups increased by approximately 1.5- or 2.1-fold, respectively, as compared to sham-injured animals (*n* = 7, Fig. 6a, b). Notably, CR8 treatment (*n* = 7) significantly attenuated (TBI + Veh + HB)-induced increase of Iba-1 expression. Moreover, immunohistochemical analysis demonstrated that TBI + Veh + HB (*n* = 6), in contrast to TBI-alone tissue (*n* = 6), caused a 1.4-fold of the total number of Iba-1^+^ microglia/macrophages at 30 days post-injury in both the ipsilateral cortex and hippocampus (Fig. 6c–e). CR8-treated rats (*n* = 6) showed significantly reduced total numbers of Iba-1^+^ cells.

**Fig. 7:**
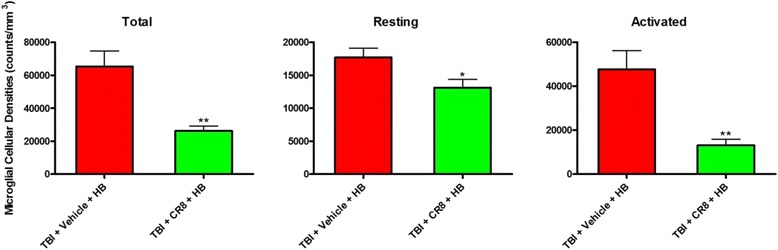
Microglial densities in the injured cortex were determined using unbiased stereological quantifications. CR8 treatment significantly reduced the total number of microglia and the number of resting and activated microglia in comparison to the TBI + Veh + HB group. *N* = 6 (TBI/Veh/HB), 6 (TBI/HB/CR8). *p < 0.05, TBI/HB/CR8 vs. TBI/Veh/HB; **p < 0.01, TBI/HB/CR8 vs. TBI/Veh/HB

It is well known the TBI significantly increases microglial activation [7–15]. In our previous study, we have shown that TBI + HB further increases activated microglia at 30 days in comparison to TBI alone [5]. In order to examine if CR8 treatment reduced the neuroinflammatory response associated with TBI + HB, stereological quantifications of resting and activated microglia cell numbers in the injured cortex were evaluated at 30 days post-HB. CR8 treatment (*n* = 6, Fig. 7) significantly reduced the total number of microglia and number of activated microglia in comparison to the TBI + HB group (*p* < 0.01, TBI + CR8 + HB vs. TBI + Veh + HB).

**Fig. 8:**
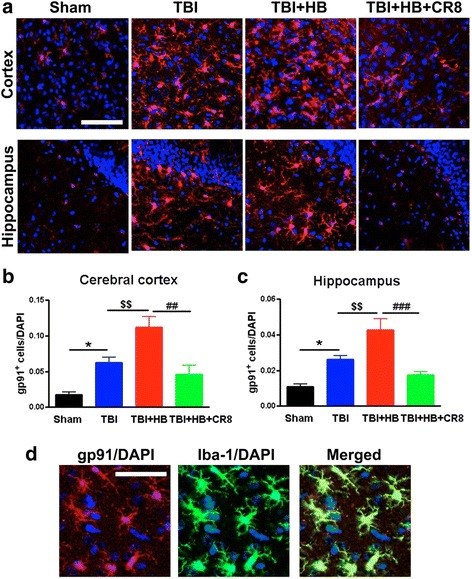
Hypobaria exposure after TBI increases the expression of NADPH oxidase membrane component gp91^phox^. **a** Representative immunofluorescent staining for gp91^phox^ (*red*) and DAPI (*blue*) in the ipsilateral cortex and hippocampus at 30 days after TBI. **b**, **c** Quantification of gp91^phox^-positive cells revealed significant increases in the TBI + Veh + HB group in contrast to the TBI-alone tissue. A significant decrease of gp91^phox^-positive cells was observed in the CR8 treatment group in comparison to the TBI/Veh/HB group. *N* = 4 (sham), 6 (TBI), 6 (TBI/Veh/HB), 6 (TBI/CR8/HB). *p < 0.05, TBI vs. sham injury; ^$$^ *p* < 0.01, TBI/Veh/HB vs. TBI; ^##^ *p* < 0.01, ^###^ *p* < 0.001, TBI/CR8/HB vs. TBI/Veh/HB. **d** gp91^phox^-positive cells (*red*) in the injured cortex were co-labeled with Iba-1^+^ microglia/macrophages (*green*; DAPI, *blue*). *Scale bar* = 50 μm in **a** and 25 μm in **d**

In addition, we evaluated the effect of HB on the expression of NADPH oxidase membrane component gp91^phox^ after TBI. Immunohistochemistry at 30 days post-injury demonstrated that TBI + Veh + HB (*n* = 6) significantly increased the total numbers of gp91^phox^-positive cells in contrast to TBI-alone tissue (*n* = 6, Fig. 8a–c). Moreover, double-labeling immunohistochemistry revealed that large numbers of gp91^phox^-positive cells in the injured coronal sections were colabeled with Iba-1 (Fig. 8d). Notably, there were fewer gp91^phox^-positive cells in the CR8-treated TBI + HB samples (*n* = 6), and Iba-1 expression was also reduced in these cells.

**Fig. 9:**
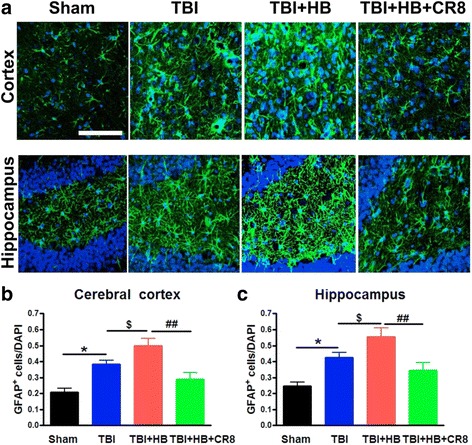
Cell cycle inhibition reduces protein expression of astrocytes marker GFAP induced by hypobaria exposure following TBI. **a** Representative immunofluorescent staining for GFAP (*green*) and DAPI (*blue*). **b**, **c** Quantification of GFAP^+^ cells revealed significant increases in the TBI + Veh + HB group in contrast to the TBI-alone tissue. A significant decrease of GFAP^+^ cells was observed in the CR8 treatment group in comparison to the TBI/HB group. *N* = 4 (sham), 6 (TBI), 6 (TBI/Veh/HB), 6 (TBI/CR8/HB). *p < 0.05, TBI vs. sham injury; ^$^ *p* < 0.05, TBI/Veh/HB vs. TBI; ^###^ *p* < 0.001, TBI/CR8/HB vs. TBI/Veh/HB. *Scale bar* = 50 μm

Quantitative immunofluorescence image analysis also showed significant increases in the total numbers of GFAP^+^ astrocytes in the TBI + Veh + HB group (*n* = 6) in contrast to the TBI-alone tissue (*n* = 6, *P* < 0.01; Fig. 9). Notably, there were significant reductions in the positively stained cells in both the ipsilateral cortex and hippocampus in CR8-treated animals (*n* = 6, *P* < 0.01).

### Cell cycle inhibition by CR8 improves functional outcomes following TBI + HB

**Fig. 10:**
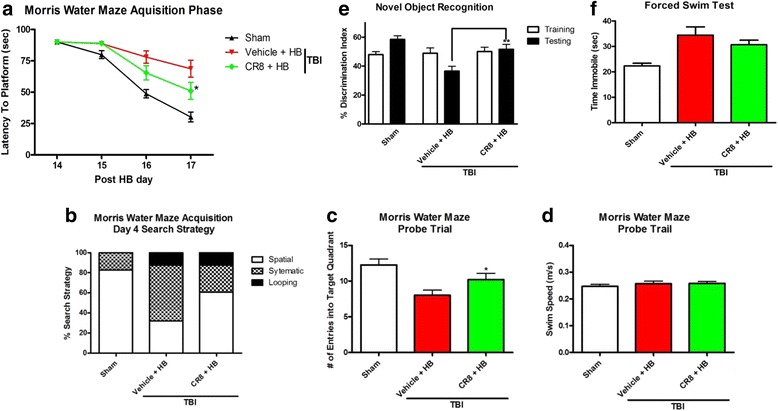
Cell cycle inhibition by CR8 improves functional outcomes following TBI plus hypobaria. **a**–**d** Cognitive assessment of CR8 treatment using the Morris water maze (MWM). The TBI + CR8 + HB group showed significant improvements in spatial learning deficits in comparison to the TBI + vehicle + HB group following prolonged hypobaria exposure at 6 h after TBI. The swimming patterns during all trials on the fourth day of the acquisition phase were analyzed to assess the search strategies utilized by the animals to locate the hidden platform. A chi-square analysis was used to compare strategies between groups and was found to be significant (*p* < 0.0001, *χ* ^2^ = 57.79, *df* = 4). Animals in the vehicle + HB group were less efficient in their search strategy while attempting to locate the hidden platform. CR8 treatment increased the percentage of trials in which a spatial search strategy was utilized. Spatial memory was assessed using the MWM probe trial on day 18 after HB by examining the number of entries into the target quadrant. CR8 treatment increased the number of target quadrant entries in comparison to the TBI + Veh + HB group indicating a reduction in retention memory deficits in the probe trial. Swim speeds did not differ across groups (*p* = 0.4382). **e** Nonspatial memory was assessed using the novel object recognition test on post-HB day 21. Animals showed an equal preference for the two identical objects during the training phase. CR8 treatment significantly increased the discrimination index in comparison to the TBI + Veh + HB group indicating an improvement in nonspatial memory. **f** Depressive-like behaviors were assessed using the forced swim test. CR8 treatment did not significantly reduce the depressive-like behavior caused by TBI plus HB. *N* = 16 (sham), 14 (TBI + Veh + HB), 15 (TBI + CR8 + HB). *p < 0.05, **p < 0.01, TBI + CR8 + HB vs. TBI + Veh + HB

The MWM was used to evaluate if CR8 treatment attenuates deficits in spatial learning caused by HB exposure following TBI (Fig. 10a). The factors of “post-injury days” (*F*(3126) = 102.803; *p* < 0.001) and “groups” (*F*(2126) = 12.576; *p* < 0.001) were found to be significant. The interaction of “post-injury days × groups” (*F*(6126) = 7.730; *p* < 0.01; repeated measures two-way ANOVA) was significant. Further analysis of differences between groups for each trial day was conducted using a one-tailed unpaired Student’s *t* test. The TBI + CR8 + HB group showed significant improvement in the spatial learning deficits in comparison to the TBI + Veh + HB group on trial day 4 (Fig. 10a: *p* < 0.05). The mean escape latency on the last day of training was 30.2 ± 3.9 s for the sham-injured group, 68.6 ± 6.7 for the Veh + HB group, and 51.0 ± 6.7 for the CR8-treated group.

The swimming patterns during all trials on the fourth day of the acquisition phase were analyzed to assess the search strategies utilized by the animals to locate the hidden platform (Fig. 10b). A chi-square analysis was used to compare strategies between groups (*p* < 0.001; *χ* ^2^ = 57.79, *df* = 4). HB-exposed animals were less efficient in their search strategy while attempting to locate the hidden platform with only 32% of the day 4 trials reflecting a spatial strategy. CR8-treated animals utilized a spatial search strategy on day 4 in a higher percentage of trials 61% than untreated injured animals. Spatial memory was assessed using the MWM probe trial on day 18 after HB by examining the number of entries into the target quadrant (Fig. 10c). CR8 treatment increased the number of target quadrant entries in comparison to the TBI + Veh + HB group indicating a reduction in spatial memory deficits in the probe trial (*p* < 0.05 vs. Veh + HB). Swim speeds did not differ across groups (Fig. 10d; *p* = 0.4382).

Nonspatial memory was assessed using the novel object recognition test on post-HB day 21 to evaluate if CR8 treatment improves non-hippocampal-dependent memory (Fig. 10e). Animals showed an equal preference for the two identical objects during the training phase. CR8 treatment significantly increased the discrimination index in comparison to the TBI + vehicle + HB group indicating an improvement in nonspatial memory (*p* < 0.01 vs. TBI + Veh + HB).

The forced swim test was performed on post-HB day 26 to determine if CR8 treatment reduces depressive-like behaviors induced by TBI + HB (Fig. 10f). CR8 treatment did not significantly reduce the depressive-like behavior caused by TBI + HB (*p* > 0.05 TBI + Veh + HB vs. TBI + CR8 + HB).

### CR8 treatment reduced lesion volume induced by hypobaria exposure following TBI

**Fig. 11:**
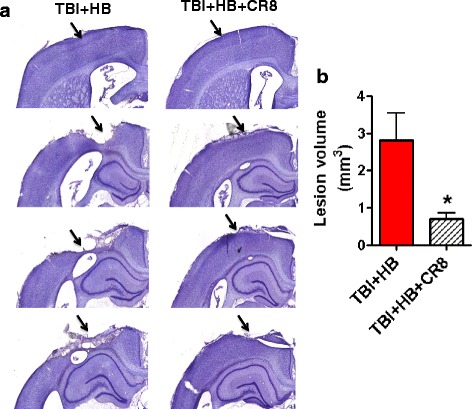
CR8 treatment reduces lesion volume induced by hypobaria exposure following TBI. Stereological assessment of lesion volume was performed at 30 days post-injury. **a** Representative images from each group are shown. Lesion cavities are marked by *arrows*. **b** There was a significant reduction in lesion volume in the TBI + CR8 + HB group when compared with the TBI + Veh + HB group. *N* = 5/group. *p < 0.05, TBI + CR8 + HB vs. TBI + Veh + HB

TBI-induced lesion volume was measured by unbiased stereological techniques (Fig. 11). Histological assessment showed that CR8 treatment (0.71 ± 0.17 mm^3^, *n* = 5) resulted in a significant reduction in lesion size after injury as compared with TBI + Veh + HB (2.8 ± 0.74 mm^3^; *n* = 5; *P* < 0.05).

### Loss of hippocampal neurons and cell cycle inhibition following TBI/HB

**Fig. 12:**
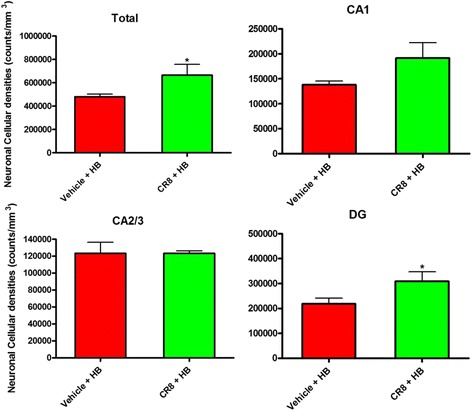
Effect of CR8 treatment following TBI plus hypobaria on neuronal cell loss in the hippocampus. Total neuronal cell numbers in the hippocampus ipsilateral to the site of injury were evaluated at 30 days post-injury. Unbiased stereological quantifications show that treatment with CR8 increased total neuronal density in the hippocampus and in the DG subregion compared with the TBI + Veh + HB group. *N* = 5 (TBI/Veh + HB), 5 (TBI/CR8/HB). * *p* < 0.05 TBI + CR8 + HB vs. TBI + Veh + HB

It is well known the TBI causes hippocampal neuronal loss [7–15]. In our previous study, we have shown that TBI + HB further increases hippocampal neuronal loss at 30 days in comparison to TBI alone [5]. Here, we evaluated whether improvements in cognitive memory function following CR8 treatment were associated with increased hippocampal neuronal survival and total neuronal cell numbers in the ipsilateral hippocampus at post-HB day 30 (Fig. 12). The CR8-treated TBI/HB group showed a significant increase in the total number of surviving hippocampal neurons and number of surviving neurons in the hippocampal DG region compared to the TBI + vehicle + HB group (*p* < 0.05).

## Discussion

Only two prior experimental studies have evaluated the effects of hypobaria following rodent TBI. We recently reported that hypobaria during simulated AE in a rat TBI model, even with oxygen levels maintained in the normal physiological range, worsens cognitive outcome and increases progressive delayed neurodegeneration and associated chronic inflammatory responses [5]. The other study showed that hypobaric exposure increased cytokine levels acutely after mouse TBI, but that group did not control for the hypoxic effects of hypobaria [6]. The present study is the first to evaluate treatment to limit the negative consequences of post-traumatic hypobaria in an animal model that simulates AE following TBI. Treatment with the CDK inhibitor CR8 reduced hypobaric-induced increases in CCA, post-traumatic microglial activation, and neurodegeneration.

CCA in the brain has been well demonstrated experimentally in models of TBI [11–15], spinal cord injury [21, 24–29], stroke [30, 31], and Alzheimer’s disease (AD) [32–35]; it has also been reported in clinical AD [36, 37]. Both neurons and glial cells show increased expression after CNS injury, and such changes may persist for weeks to months [8–10]. Chronic proliferation/activation of astrocytes and microglia resulting from CCA may contribute to secondary injury and limit neurorestoration [15, 18]. Reactive astrocytes are involved in the formation of the glial scar, which can inhibit axonal regeneration [38]. Activated microglia can produce pro-inflammatory cytokines and ROS, leading to neuronal degeneration [18]. Adult differentiated post-mitotic neurons can re-enter the cell cycle; however, such re-entry is associated with caspase-mediated neuronal apoptosis [11, 39–41].

CCA is an intricate and highly regulated process [8–10]. Through each phase in the cycle, there is a systematic progression of synthesis and degradation of phase-specific cyclin proteins. Cyclins bind and activate Ser/Thr kinases known as cyclin-dependent kinases (CDKs), which in turn phosphorylate additional substrates that promote transcription of other cyclins and progression through the cycle. During the first phase of the cell cycle, Gap 1 (G1), levels of cyclin D increase. Cyclin D binds to CDK4, promoting phosphorylation of the retinoblastoma (Rb) family of proteins. Phosphorylated Rb proteins dissociate from E2F transcription factors, which translocate to the cell nucleus and induce the transcription of other cyclins. Proteins involved in the G1 phase of the cell cycle have been identified as part of a pro-apoptotic pathway in post-mitotic neurons [9, 10]. Activation of CDK4 by cyclin D1 has been found to be necessary for apoptosis in neurons that re-enter the cell cycle [39, 41]. Furthermore, reduction of CDK4 expression is protective against apoptosis in primary neuronal cell cultures and ablation of the cyclin D1 gene limits lesion development and improves functional outcomes following TBI [42, 43].

Treatment with cell cycle inhibitors increases neuronal survival and reduces glial proliferation/activation in several CNS injury models, including rat and mouse TBI models [11–15, 21, 25–29]. After fluid percussion injury, administration of the pan-CDK inhibitor flavopiridol reduced cyclin D1 expression in neurons and glia in the cortex and hippocampus; treatment also decreased neuronal cell death, lesion volume, astroglial scar formation, and microglial activation and improved motor and cognitive recovery [11]. Central administration of another CDK inhibitor, roscovitine, 30 min after fluid percussion-induced brain injury, significantly decreased lesion volume, as well as improving motor and cognitive recovery [12]. Roscovitine also attenuated neuronal death and inhibited activation of CCA in neurons, as well as decreasing microglial activation and astrogliosis. In primary cortical microglial and neuronal cultures, roscovitine treatment attenuated neuronal cell death and decreased microglial activation as well as microglial-dependent neurotoxicity [12]. Central administration of CR8, a selective and highly potent CDK inhibitor structurally related to roscovitine, attenuated CCA pathways, and reduced post-traumatic apoptotic cell death at 24 h post-TBI [14, 15]. Administration of CR8 at 3 h post-injury limited CCA, reduced microglial activation and lesion volume, and improved behavioral outcomes in both mouse and rat models of experimental TBI [14, 15].

In the present study, the cell cycle markers cyclin D1, PCNA, and CDK4 were significantly increased following TBI/HB at 24 h and 30 days compared to TBI alone. It is well known that the upregulation of cell cycle proteins occurs in both post-mitotic cells (neurons, mature oligodendroglia) and proliferating cell types including microglia and astrocytes after TBI. In addition to neurons, microglia, and astrocytes that are predominant cell types in the brain, mature oligodendrocytes also undergo apoptotic cell death at acute phase post-injury (e.g., d1, d3 post-injury). We demonstrate changes across brain cell types [11]. We have shown that CCA inhibitors (flavopiridol, CR8) significantly decrease oligodendroglial apoptosis in the injured spinal cord at 1 day post-injury [24, 26]. Whether or not hypobaria (HB) in TBI animals increases CCA expression in oligodendrocytes is intriguing, that merits further research. In addition, expression levels of the microglial/macrophage markers Iba-1 and gp91^phox^ and the astroglial marker GFAP were significantly higher in the TBI/HB group than in TBI animals without HB. Treatment with CR8 prior to HB exposure significantly reduced the expression of cell cycle, microglia/macrophage, and astrocytes markers in comparison to the vehicle-treated TBI/HB group. CR8 significantly reduced deficits in spatial learning and retention memory function caused by HB exposure plus TBI, as reflected by the MWM and novel object recognition tests. However, CR8 treatment did not reduce depressive-like behavioral changes associated with TBI + HB exposure. Chronic microglial activation at 30 days after TBI/HB was also attenuated by CR8 treatment. There was no significant difference between TBI/HB/CR8 and TBI-alone groups in any of the assays performed. Although CR8 treatment at current dose and timing of administration did not completely reverse CDK4 signaling in the injured cortex (*p* < 0.05, TBI/HB/CR8 vs. sham) at both acute and chronic time points post-injury, the other CCA components tested (cyclin D1, PCNA) as well as markers for inflammation (Iba-1, gp91) and astrogliosis (GFAP) were significantly reduced by CR8 treatment to close to a basal level. There was no significant difference between TBI/HB/CR8 and sham groups in the ipsilateral cortex and hippocampus at day 1 or days 28 post-injury. Thus, CR8-mediated reduction of CCA and neuroinflammation are associated with improved functional outcome after HB exposure in TBI animals. In the present study, CR8 dose and timing of administration were based on previous studies using this compound in experimental animal models of TBI—which have shown neuroprotection by limiting microglial activation, astrocytosis, and neuronal loss. However, optimizing CR8 treatment condition is intriguing which merits further research. Collectively, these results suggest that increases in CCA activation caused by HB exposure following TBI result in neuronal cell death and neurotoxic microglial activation, which likely contribute to the exacerbation of cognitive dysfunction. CR8 treatment attenuated CCA and neuroinflammatory responses that were intensified by HB exposure following TBI. Given that changes in AE procedures and timing may not be possible because of the need to rapidly transport critically ill patients to definitive treatment sites, use of cell cycle inhibitors—previously examined in late human phase cancer treatment studies—should be explored as a way of limiting the negative consequences of AE in TBI patients.

## Conclusions

Wartime casualties with traumatic brain injuries may be exposed to prolonged periods of hypobaria during aeromedical evacuation. As hypobaria exposure following TBI has been shown to worsen pathophysiological and cognitive outcomes, treatments to mitigate these effects must be identified. This study evaluated the effects of prolonged hypobaria in rats subjected to traumatic brain injury on cell cycle and neuroinflammatory pathways and examined the ability of the cell cycle inhibitor CR8 to alleviate the associated effects. Hypobaria exposure following TBI significantly increased markers of cell cycle activation and microglial activation. These changes were limited by treatment with the cell cycle inhibitor CR8 at 3 h post-injury. CR8 treatment also significantly reduced cognitive deficits associated with hypobaria exposure following injury.

## Abbreviations

AD: Alzheimer’s disease
AE: aeromedical evacuation
CA1, CA2, CA3: cornus ammonis 1, 2, 3
CCA: cell cycle activation
D.I.: discrimination index
DAPI: 4′,6-diamidino-2-phenylindole
DG: dentate gyrus
FP: fluid percussion
HB: hypobaria
HRP: horseradish peroxidase
Iba-1: ionized calcium-binding adapter molecule 1
MWM: Morris water maze
PCNA: proliferating cell nuclear antigen
Rb: retinoblastoma
RIPA: radioimmunoprecipitation assay
ROS: reactive oxygen species
SDS: sodium dodecyl sulfate
TBI: traumatic brain injury

## References

[1] Faul M, Xu L, Waid MM, Coronado VG (2010). Traumatic brain injury in the United States: emergency department visits. Hospitalizations and deaths 2002–2006.

[2] Defense and Veterans Brain Injury Center. DOD TBI worldwide numbers since 2000. In: DOD TBI Worldwide Numbers. 2016. http://dvbic.dcoe.mil/sites/default/files/uploads/Worldwide%20Totals%202000-2014Q1.pdf. Accessed 15 June 2016

[3] Reno J (2010). Military aeromedical evacuation, with special emphasis on craniospinal trauma. Neurosurg Focus.

[4] Fang R, Dorlac G, Allan P, Dorlac W (2010). Intercontinental aeromedical evacuation of patients with traumatic brain injuries during operations Iraqi Freedom and Enduring Freedom. Neurosurg Focus.

[5] Skovira JW, Kabadi SV, Wu J, Zhao Z, DuBose J, Rosenthal R, Fiskum G, Faden AI (2016). Simulated aeromedical evacuation exacerbates experimental brain injury. J Neurotrauma.

[6] Goodman MD, Makley AT, Huber NL, Clarke CN, Friend LA, Schuster RM, Bailey SR, Barnes SL, Dorlac WC, Johannigman JA, Lentsch AB, Pritts TA (2011). Hypobaric hypoxia exacerbates the neuroinflammatory response to traumatic brain injury. J Surg Res.

[7] Faden AI (2011). Microglial activation and traumatic brain injury. Ann Neurol.

[8] Cernak I, Stoica B, Byrnes KR, Di Giovanni S, Faden AI (2005). Role of the cell cycle in the pathobiology of central nervous system trauma. Cell Cycle.

[9] Byrnes KR, Faden AI (2007). Role of cell cycle proteins in CNS injury. Neurochem Res.

[10] Stoica BA, Byrnes KR, Faden AI (2009). Cell cycle activation and CNS injury. Neurotox Res.

[11] Di Giovanni S, Movseyan V, Ahmed F, Cernak I, Schinelli S, Stoica B (2005). Cell cycle inhibition provides neuroprotection and reduces glial proliferation and scar formation after traumatic brain injury. PNAS.

[12] Hilton GD, Stoica BA, Byrnes KR, Faden AI (2008). Roscovitine reduces neuronal loss, glial activation, and neurologic deficits after brain trauma. J Cereb Blood Flow Metab.

[13] Kabadi SV, Stoica BA, Byrnes KR, Hanscom M, Loane DJ, Faden AI (2012). Selective CDK inhibitor limits neuroinflammation and progressive neurodegeneration after brain trauma. J Cereb Blood Flow Metab.

[14] Kabadi SV, Stoica BA, Hanscom M, Loane DJ, Kharebava G, Murray Ii MG, Cabatbat RM, Faden AI (2012). CR8, a selective and potent CDK inhibitor, provides neuroprotection in experimental traumatic brain injury. Neurotherapeutics.

[15] Kabadi SV, Stoica BA, Loane DJ, Luo T, Faden AI (2014). CR8, a novel inhibitor of CDK, limits microglial activation, astrocytosis, neuronal loss, and neurologic dysfunction after experimental traumatic brain injury. J Cereb Blood Flow Metab.

[16] Dardiotis E, Karanikas V, Paterakis K, Fountas K, Hadjigeorgiou GM. Traumatic brain injury and inflammation: emerging role of innate and adaptive immunity. In: Agrawal A, editor. Brain injury—pathogenesis, monitoring, recovery and management. ISBN: 978-953-51-0265-6, InTech, March 2012

[17] Colton C (2009). Heterogeneity of microglial activation in the innate immune response in the brain. J Neuroimmume Pharmacol.

[18] Loane DJ, Byrnes KR (2010). Role of microglia in neurotrauma. Neurotherapeutics.

[19] Ramlackhansingh AF, Brooks DJ, Greenwood RJ, Bose SK, Turkheimer FE, Kinnunen KM, Gentleman S, Heckemann RA, Gunanayagam K, Gelosa G (2011). Sharp inflammation after trauma: microglial activation and traumatic brain injury. J Ann Neurol.

[20] Kabadi SV, Hilton GD, Stoica BA, Zapple DN, Faden AI (2010). Fluid-percussion-induced traumatic brain injury model in rats. Nat Protoc.

[21] Wu J, Zhao Z, Sabirzhanov B, Stoica BA, Kumar A, Luo T, Skovira J, Faden AI (2014). Spinal cord injury causes brain inflammation associated with cognitive and affective changes: role of cell cycle pathways. J Neurosci.

[22] Sarkara C, Zhaoa Z, Aungst S, Sabirzhanova B, Faden AI, Lipinski MM (2014). Impaired autophagy flux is associated with neuronal cell death after traumatic brain injury. Autophagy.

[23] Liu S, Sarkar C, Dinizo M, Faden AI, Koh EY, Lipinski MM, Wu J (2015). Disrupted autophagy after spinal cord injury is associated with ER stress and neuronal cell death. Cell Death Dis.

[24] Byrnes KR, Stoica BA, Fricke S, Di Giovanni S, Faden AI (2007). Cell cycle activation contributes to post-mitotic cell death and secondary damage after spinal cord injury. Brain.

[25] Tian DS, Xie MJ, Yu ZY, Zhang Q, Wang YH, Chen B, Chen C, Wang W (2007). Cell cycle inhibition attenuates microglia induced inflammatory response and alleviates neuronal cell death after spinal cord injury in rats. Brain Res.

[26] Wu J, Stoica BA, Dinizo M, Pajoohesh-Ganji A, Piao C, Faden AI (2012). Delayed cell cycle pathway modulation facilitates recovery after spinal cord injury. Cell Cycle.

[27] Wu J, Pajoohesh-Ganji A, Stoica BA, Dinizo M, Guanciale K, Faden AI (2012). Delayed expression of cell cycle proteins contributes to astroglial scar formation and chronic inflammation after rat spinal cord contusion. J Neuroinflammation.

[28] Wu J, Stoica BA, Luo T, Sabirzhanov B, Zhao Z, Guanciale K, Nayar SK, Foss CA, Pomper MG, Faden AI (2014). Isolated spinal cord contusion in rats induces chronic brain neuroinflammation, neurodegeneration, and cognitive impairment. Involvement of cell cycle activation. Cell Cycle.

[29] Wu J, Zhao Z, Zhu X, Renn CL, Dorsey SG, Faden AI (2016). Cell cycle inhibition limits development and maintenance of neuropathic pain following spinal cord injury. Pain.

[30] Rashidian J, Iyirhiaro GO, Park DS (2007). Cell cycle machinery and stroke. Biochim Biophys Acta.

[31] Osuga H, Osuga S, Wang F, Fetni R, Hogan MJ, Slack RS, Hakim AM, Ikeda JE, Park DS (2000). Cyclin-dependent kinases as a therapeutic target for stroke. Proc Natl Acad Sci U S A.

[32] Sanphui P, Pramanik SK, Chatterjee N, Moorthi P, Banerji B, Biswas SC (2013). Efficacy of cyclin dependent kinase 4 inhibitors as potent neuroprotective agents against insults relevant to Alzheimer’s disease. PLoS One.

[33] Seward ME, Swanson E, Norambuena A, Reimann A, Cochran JN, Li R, Roberson ED, Bloom GS (2013). Amyloid-β signals through tau to drive ectopic neuronal cell cycle re-entry in Alzheimer’s disease. J Cell Sci.

[34] Arendt T (2012). Cell cycle activation and aneuploid neurons in Alzheimer’s disease. Mol Neurobiol.

[35] Neve RL, McPhie DL (2006). The cell cycle as a therapeutic target for Alzheimer’s disease. Pharmacol Ther.

[36] Katsel P, Tan W, Fam P, Purohit DP, Haroutunian V (2013). Cell cycle checkpoint abnormalities during dementia: a plausible association with the loss of protection against oxidative stress in Alzheimer’s disease. PLoS One.

[37] Kim H, Kwon Y-A, Ahn IS, Kim S, Kim S, Ahn Jo S, Kim DK (2016). Overexpression of cell cycle proteins of peripheral lymphocytes in patients with Alzheimer’s disease. Psychiatry Investig.

[38] Silver J, Miller JH (2004). Regeneration beyond the glial scar. Nat Rev Neurosci.

[39] Kranenburg O, van der Eb AJ, Zantema A (1996). Cyclin D1 is an essential mediator of apoptotic neuronal cell death. EMBO J.

[40] Herrup K, Yang Y (2007). Cell cycle regulation in the postmitotic neuron: oxymoron or new biology?. Nat Rev Neurosci.

[41] Shan B, Lee WH (1994). Deregulated expression of E2F-1 induces S-phase entry and leads to apoptosis. Mol Cell Biol.

[42] Rashidian J, Iyirhiaro G, Aleyasin H, Rios M, Vincent I, Callaghan S, Bland RJ, Slack RS, During MJ, Park DS (2005). Multiple cyclin-dependent kinases signals are critical mediators of ischemia/hypoxic neuronal death in vitro and in vivo. Proc Natl Acad Sci U S A.

[43] Kabadi SV, Stoica BA, Loane DJ, Byrnes KR, Hanscom M, Cabatbat RM, Tan MT, Faden AI (2012). Cyclin D1 gene ablation confers neuroprotection in traumatic brain injury. J Neurotrauma.

